# High Chili Intake and Cognitive Function among 4582 Adults: An Open Cohort Study over 15 Years

**DOI:** 10.3390/nu11051183

**Published:** 2019-05-27

**Authors:** Zumin Shi, Tahra El-Obeid, Malcolm Riley, Ming Li, Amanda Page, Jianghong Liu

**Affiliations:** 1 Human Nutrition Department, College of Health Science, QU Health, Qatar University, Doha 2713, Qatar; tahra.e@qu.edu.qa; 2 Commonwealth Scientific and Industrial Research Organisation (CSIRO), Adelaide, SA 5000, Australia; malcolm.riley@csiro.au; 3 Centre for Population Health Research, Division of Health Sciences, University of South Australia; Adelaide, SA 5000, Australia; Ming.Li@unisa.edu.au; 4 Adelaide Medical School, University of Adelaide, Adelaide, SA 5000, Australia; amanda.page@adelaide.edu.au; 5 School of Nursing, University of Pennsylvania, Philadelphia, PA 19104, USA; jhliu@nursing.upenn.edu

**Keywords:** Chili intake, cognitive function, obesity, Chinese, adults

## Abstract

We aimed to examine the association between chili intake and cognitive function in Chinese adults. This is a longitudinal study of 4852 adults (age 63.4 ± 7.7) attending the China Health and Nutrition Survey during 1991 and 2006. Cognitive function was assessed in 1997, 2000, 2004 and 2006. In total, 3302 completed cognitive screening tests in at least two surveys. Chili intake was assessed by a 3-day food record during home visits in each survey between 1991 and 2006. Multivariable mixed linear regression and logistic regression were used. Chili intake was inversely related to cognitive function. In fully adjusted models, including sociodemographic and lifestyle factors, compared with non-consumers, those whose cumulative average chili intake above 50 g/day had the regression coefficients (and 95% CI) for global cognitive function of −1.13 (−1.71–0.54). Compared with non-consumers, those with chili consumption above 50 g/day had the odds ratio (and 95% CI) of 2.12(1.63–2.77), 1.56(1.23–1.97) for self-reported poor memory and self-reported memory decline, respectively. The positive association between chili intake and cognitive decline was stronger among those with low BMI than those with high BMI. The longitudinal data indicate that higher chili intake is positively associated with cognitive decline in Chinese adults in both genders.

## 1. Introduction

Dementia is a common disease that affects the quality of life, especially among the elderly population. The prevalence of dementia is on the rise. It is estimated that dementia affected 35.6 million people worldwide in 2010, and this number is expected to double every 20 years [1]. In 2017, approximately 9.5 million Chinese adults aged 60 years and above had dementia [2]. Dietary factors are among the many modifiable risk factors (e.g., lower education, hypertension, obesity, diabetes, smoking, depression and physical inactivity [3]) for dementia [4].

Chili is one of the most commonly used spices in the world [5] with a substantial higher chili intake in Asian compared to European countries [6]. In certain regions of China such as Sichuan and Hunan, almost one in three adults consume spicy food, including chili, daily [7]. Recent epidemiological studies suggest that chili consumption is inversely related to mortality [7], obesity [8], and hypertension [9]. It has been hypothesized that the beneficial effects of chili are due to its active component, capsaicin. Capsaicin can activate the ion channel transient receptor potential vanilloid subtype 1 (TRPV1) leading to inhibition of vascular oxidative stress [10], reduced energy intake, increased energy expenditure and enhanced fat oxidation [8,11,12,13,14].

In animal studies, the role of capsaicin in cognitive function is inconclusive. Some studies suggest that capsaicin is beneficial for cognitive function [15] or Alzheimer’s disease [16], while other studies found capsaicin to be neurotoxic [17,18]. Epidemiological studies on the association between chili consumption and cognitive function are limited. Currently, only one study has assessed the association between chili consumption and cognitive function [19]. In this cross-sectional study, Liu et al. reported that dietary capsaicin intake was positively associated with cognitive function in middle-aged and elderly Chinese [19]. Further investigation is warranted to explore these findings. Moreover, the long-term effects of chili on cognition are unclear.

We have previously reported that chili consumption was inversely associated with the risk of obesity [8] and hypertension [9]. It is well known that hypertension adversely affects cognitive function [20]. In contrast, obesity has been shown to be positively associated with cognitive function in some observational studies [21,22]. Whether the benefits of chili intake interplay with other factors, such as obesity and hypertension, to affect cognitive function is not clear.

In the current study, we aimed to assess the longitudinal association between chili intake and cognitive function among Chinese adults using data collected over a 15-year period from the China Health and Nutrition Survey (CHNS). The second aim was to assess the interaction between chili intake, BMI/hypertension in relation to cognitive function. The third aim was to explore the cross-sectional relationship between chili intake and cognitive function. We hypothesized that chili intake is inversely associated with cognitive function decline.

## 2. Materials and Methods

### 2.1. Study Design and Sample

**Figure 1:**
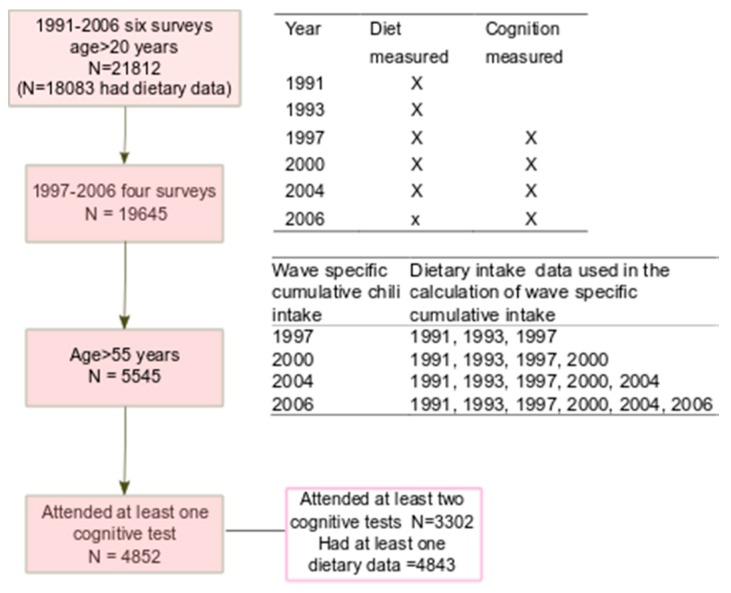
Sample flowchart of participants attending the China Health and Nutrition Survey. The table in the top right corner shows the measures of diet and cognition conducted in each survey. The middle table shows the calculation of wave specific cumulative chili intake.

The CHNS study is an ongoing open prospective household-based cohort study conducted in nine provinces in China between 1989 and 2011 [23,24]. Participants may join or leave the study in any survey wave. CHNS uses a multistage random-cluster sampling process to select samples in both urban and rural areas. Nine waves of data collection (i.e., 1989, 1991, 1993, 1997, 2000, 2004, 2006, 2009, and 2011) have been conducted. Cognitive screen tests were conducted among those above the age of 55 in 1997, 2000, 2004 and 2006 surveys. Dietary intake was assessed in each survey between 1991 and 2006. In total, 4852 participants (2309 men and 2543 women) attended the cognitive screen tests between 1997 and 2006 (Figure 1). The mean age of the participants attending the first cognitive function test was 63.4 ± 7.7 years. Of these participants, 3302 attended the screen test in at least two surveys. Participants who completed at least one cognitive screen test were included in the analysis. No participants were excluded due to missing values for diet or cognition.

The survey was approved by the institutional review committees of the University of North Carolina (USA) and the National Institute of Nutrition and Food Safety (China). Informed consent was obtained from all participants. The response rate based on those who participated in 1989 and remained in the 2006 survey was >60%.

### 2.2. Outcome Variable: Cognitive Function

The global cognitive score was calculated using composite scores of memory, counting back and subtraction scores. The face-to-face cognitive screening items used in CHNS included a subset of items from the Telephone Interview for Cognitive Status—modified [25]. This tool has been used in other population studies in China to assess cognitive function [26]. Cognitive screening included immediate (score 10) and delayed recall of a 10-word list (score 10), counting backward from 20 (score 2), and serial 7 subtraction (score 5). A total verbal memory score was constructed as the sum of the immediate and delayed 10-word recall. An orientation test was not included in the analysis as it was only assessed in 1997, 2000, and 2004. The total global cognitive score ranged from 0–27. The cognitive function test started with the immediate recall of a 10-word list. The interviewer (i.e., trained health worker) read ten words at a speed of two seconds per word. The participants were given two minutes to memorize the ten words. For each correct recalled word, a score of 1 was given. The participants were then asked to count back from 20 to 1. If the participants made a mistake in the first try, a second chance was given. A score of 2 was given to those answered correctly in the first try, or 1 in the second try. After the count test, the participants were asked to do five consecutive subtractions of 7 from 100. Each correct subtraction was given a score of 1. Finally, the participants were asked to recall the 10-word list tested before. Each recalled word was given a score of 1. A high cognitive score represents better functioning cognition.

Participants were also asked “How is your memory? (1) Very good; (2) good; (3) OK; (4) bad; (5) very bad; (9) unknown”. Those who reported “bad” or “very bad” were defined as having a poor memory. Memory change was assessed by the question “In the past twelve months, how has your memory changed? (1) Improved; (2) stayed the same; (3) declined; (9) unknown”. Those who reported “declined” were defined as self-reported memory decline.

### 2.3. Exposure Variable: Cumulative Mean Chili Intake

Derived from dietary surveys, chili intake included both fresh and dried chili peppers, but did not include sweet capsicum or black pepper. Cumulative mean chili intake was calculated for each individual at each survey from all the proceeding years of chili intake in order to reduce variation within individuals and to represent long term habitual intake [27]. Details on wave-specific cumulative chili intake are described in Figure 1. For example, the cumulative average intake of chili in 2004 was the mean intake of chili in 1991, 1993, 1997, 2000 and 2004.

Detailed description of the dietary measurement has been published previously [23]. In short, at each wave, individual dietary intake data were collected by a trained investigator conducting a 24h dietary recall on each of 3 consecutive days. Food and condiments in the home inventory, food purchased from markets or picked from gardens, and food waste were weighed and recorded by interviewers at the beginning and end of the three-day survey period. Food consumption data were converted to nutrient intake using the Chinese Food Composition Table [28]. The dietary assessment method has been validated for energy intake [29]. The overall chili intake for mixed dishes was estimated based on the fresh ingredients.

Height, weight, and blood pressure were measured at each wave. Overweight/obesity was defined as BMI ≥ 24 kg/m^2^. Hypertension was defined as systolic blood pressure above 140 mmHg and/or diastolic blood pressure above 90 mmHg, or having known hypertension.

### 2.4. Covariates

In the analyses we treated the following variables as covariates: Education (low: Illiterate/primary school; medium: Junior middle school, and high: High middle school or higher), per capita annual family income (recoded into tertiles as low, medium and high), urbanization levels [23] (recoded into tertiles as low, medium and high), physical activity level (metabolic equivalent of task, (MET)), smoking (non-smokers, ex-smokers and current smokers), alcohol drinking (yes or no) and self-reported diabetes and stroke (yes or no).

Two dietary patterns were constructed based on 35 food groups, including alcohol, aggregated from 3-day food records using factor analysis [30]. The first pattern (traditional south pattern) is characterized by a high intake of rice, pork, and vegetables, and low intake of wheat; the second pattern (modern dietary pattern) had a high intake of fruit, soy milk, egg, milk, deep fried products and beer.

### 2.5. Data Analyses

Cumulative mean chili intake was recoded into four groups: Non-consumers, 1–20 g/day, 20–50 g/day and >50 g/day. We choose this cut off based on our previous paper as well as the serving size [9]. The serving size in the context of Chinese food is a Liang (1 Liang =50 g). The median portion size of chili intake was 50 g. Furthermore, among the chili consumers, about 30% had chili intake above 50 g per day [9]. The chi-square test was used to compare differences between groups for categorical variables and ANOVA for continuous variables. Due to the open cohort study design, following common practice and making use of all available data, we did not exclude those who had only one measure of cognitive function test in the overall analysis. However, these participants were excluded from the final multivariable model. In Stata 15.1, a mixed effect model using mixed command was used to assess the association between chili intake and cognitive function. A negative regression coefficient represents cognitive function decline. A set of models were used: Model 1 adjusted for age, gender and energy intake; model 2 further adjusted for intake of fat, smoking, alcohol drinking, income, urbanization, education, and physical activity; model 3 further adjusted for dietary patterns; model 4 further adjusted for BMI and hypertension; model 5 further excluded those who only participated in one wave of the cognitive tests. A linear trend was tested after modelling the median intake to the chili intake levels as a continuous variable in a regression model. Models 1–4 were used to assess the cross-sectional association between chili intake and cognition as they included participants who attended only way survey.

A mixed-effect logistic regression adjusting for covariates, the same as model 4 mentioned above, was used to assess the association between cumulative mean chili intake and the risk of self-reported poor memory and memory decline. To test the interaction between chili intake and BMI, hypertension, gender, urbanization and income, a product term of these two variables was put into the regression model. Chili intake level (0, 1, 2, 3) was treated as a continuous to test the interaction. We used *marginsplot* command in Stata 15.1 to visually present the interaction between chili intake and BMI in relation to global cognitive score. In sensitivity analyses, we further adjusted for diabetes and stroke.

All the analyses were performed using STATA 15.1 (Stata Corporation, College Station, TX, USA). Statistical significance was considered when *p* < 0.05 (two-sided).

## 3. Results

### 3.1. Descriptive Results

The sample characteristics among participants attending the first cognitive function test based on the different levels of cumulative chili intake is illustrated in Table 1. Energy, protein and carbohydrate intake was positively associated with chili intake. However, there was no difference in fat intake across the different categories of chili intake. Participants with a high chili consumption had a lower income and BMI, and were more physically active compared with non-consumers. Traditional and modern dietary patterns were positively and inversely associated with chili consumption, respectively.

**Figure 2:**
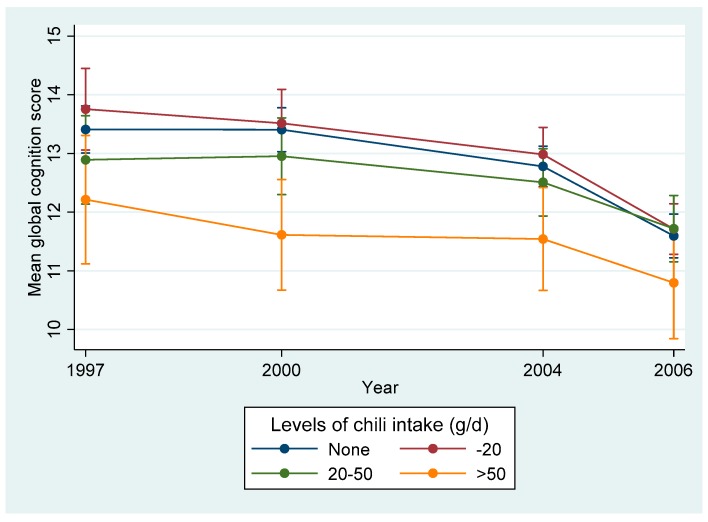
Mean global cognitive score (95% CI) by year and chili intake among Chinese adults aged >= 55 years and who attended at least two waves of cognition tests, China Health and Nutrition Survey. The numbers of participants who attended the cognitive test in 1997, 2000, 2004, and 2006 were: 1573, 2019, 2694, and 2565 respectively.

The cumulative mean chili intake was 12.5 g/day (SD 22.7) in 1997. The mean global cognition score was 12.1 (SD 6.8) in 1997. The decline in the annual cognitive function score was 0.10 (95% CI 0.07–0.13) (*p* < 0.001). Overall, the mean global cognitive score declined in all chili intake levels between 1997 and 2006 (Figure 2). The prevalence of self-reported poor memory and memory decline increased with the increase of chili intake (Figure S1).

### 3.2. Association between Chili Intake and Cognitive Function

Chili intake was positively related to cognitive function decline (Table 2). Compared with non-consumers, those who consumed more than 50 g/day had a lower global cognitive score. In fully adjusted models (model 5), those who ate chili 0, 1–20, 20–50 and >50 g/day had regression coefficients (95% CI) for the global cognitive score of 0, 0.17 (−0.14–0.52), −0.31 (−0.72–0.10) and −1.13 (−1.71–0.54) respectively. In sensitivity analyses, after further adjusting for diabetes or stroke, the above association between chili intake and cognition did not change (data not shown). Using chili intake as a continuous variable, in the fully adjusted model (model 5), for each 10 g increase of chili intake, the beta coefficient for the global cognitive score was −0.13 (95%CI −0.20, −0.07) (*p* < 0.001).

Overall, compared with non-consumers, a chili intake higher than 50 g/day was associated with more than twice the risk of having self-reported poor memory (OR, 2.12 (1.63–2.77)), and 56% increased risk of having self-reported memory decline (OR, 1.56(1.23–1.97)) (Table 3).

### 3.3. Weight Status Modifies the Association between Chili Intake and Cognitive Function

**Figure 3:**
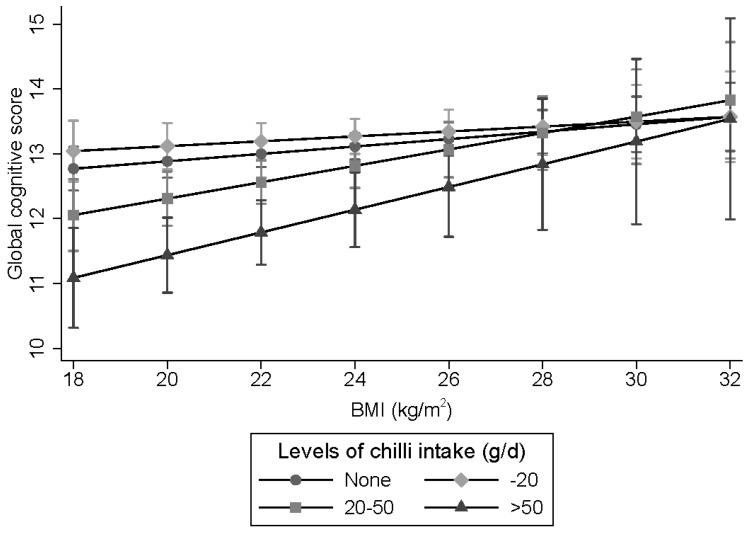
Interaction between chili intake and BMI in relation to global cognitive function. The mixed linear regression model adjusted for age, gender, intake of energy and fat, smoking, alcohol drinking, income, urbanicity, education, and physical activity, overall dietary patterns and hypertension is shown. Values represent regression coefficients and 95% CI. *p* for interaction between BMI and chili intake was 0.046. An ordinal value (0, 1, 2, 3) was assigned to reflect the chili intake level and treated as a continuous variable while testing for interactions.

A significant interaction (*p* = 0.046) between chili intake and BMI in relation to cognitive function was found (Figure 3). The effect of chili intake on cognition was stronger among those with a low BMI than high BMI. 

## 4. Discussion

In this population-based open cohort study, high chili intake was positively associated with lower cognitive scores as measured by global cognitive scores and self-reported memory loss among those who attended at least two cognitive tests between 1997 and 2006. In addition, there was a borderline significant interaction between chili intake and BMI. High chili intake was positively associated with self-reported poor memory. The positive association between chili intake and cognitive decline was stronger among those with a low BMI. The association was independent of lifestyle factors.

To our knowledge, this is the first longitudinal population study to investigate the association between chili intake and cognitive function. In contrast to the current study, a cross-sectional study of 338 Chinese adults aged 40 years and above living in a community in Chongqing, a city with high chili consumption, found that a capsaicin-rich diet was positively associated with cognitive function and inversely associated with blood amyloid-β levels [19]. However, this study used a food frequency questionnaire to collect food consumption, and there was a significant association with age for both the quantity and frequency of chili intake. Participants with a high chili intake were younger than those who did not consume chili. Furthermore, there was no association between chili consumption and BMI or hypertension in this population, and therefore, it is possible that older people in this population avoided chili consumption due to chronic disease. The discrepancy between the current study and this study may also be due to study design. Reverse causation is possible in cross-sectional studies. For example, in rodents, brain-derived neurotrophic factor (BDNF) has been shown to regulate food intake [31]. People with cognitive impairment have a lower level of BDNF than those with normal cognitive function [32,33]. It could be that low BDNF levels inhibit the preference for chili, leading to a low chili consumption.

The mechanisms linking chili intake and cognitive function decline have yet to be fully elucidated. Evidence from animal studies provides conflicting results regarding the role of capsaicin in cognition. A study suggested that red peppers with moderate and severe pungency prevent memory deficit in rats [34]. In adult rats, capsaicin administration was associated with an initial increase in BDNF levels, within 1–3 days, followed by a decrease in BDNF after 4 weeks [35]. BDNF is important for neuronal survival, growth, and differentiation, and has an important role in learning memory [36]. Alternatively, some studies suggest that capsaicin is neurotoxic [17,18] and high doses of capsaicin have been used as a tool for chemical denervation of sensory nerves [37]. Therefore, high chili consumption may impact on neuronal viability and, as a consequence, cognitive function. However, this is highly speculative and requires further investigation. Although chili intake is inversely associated with the risk of hypertension [9], this beneficial effect has not translated to a better cognitive function. Whether BMI mediates the association between chili intake and cognitive function remains to be studied. We found chili intake decreased the risk of obesity in our previous study [8]. In the current study, the effect of chili on cognitive function appears to be stronger among those with normal body weight. It could be that those with normal body weight are more sensitive to chili intake. Adiposity has been shown to be beneficial for cognitive function among middle-aged and older people [21,22]. However, some studies suggested that this could be due to a reverse causation [38]. Further research is needed to elucidate the mechanism behind the chili-cognition association.

Our study has several strengths including the longitudinal study design, multiple measurements of dietary intake including chili intake, the relatively large sample size, as well as the wide variation of chili intake and the adjustment for BMI in our analysis. The use of cumulative chili intake based on the repeated measure of 3-day dietary intake in combination with household food inventory provides a robust estimate of long-term chili intake. The study population provides a unique opportunity to assess the association between high chili intake and cognition function as the daily consumption of chili above 50 g is not common in Western countries. One of the main limitations is that we were not able to explore the potential mechanisms due to a lack of related biomarkers. Although we have adjusted for potential confounders, including sociodemographic and lifestyle factors, residual confounding factors may still impact our results. For example, it is well known that level of education affects cognitive function. In our study, there was a significant difference in chili intake among people with different education levels. Therefore, it is possible that the confounding effect of education may still contribute to the relationship between chili intake and cognitive function. Future random control trial designs may help tease out this complicated relationship to confirm chili intake and cognitive function.

In conclusion, high chili intake was associated with cognitive decline among Chinese adults, especially among those with normal body weight. The findings do not support high chili consumption in older people. While we observed that chili intake above 50 g/day was associated with almost doubled the risk of self-reported poor memory, further research is needed to test whether reducing chili intake can prevent cognitive decline.

## Figures and Tables

**Table 1:** Sample characteristics of Chinese adults aged ≥55 years old attending the first cognitive function test by levels of cumulative chili intake (*n* = 4661) ^1^.

	None	1–20	20–50	>50	*p*-value
*n*	2662	975	718	346	
Age (years)	64.3 (7.9)	62.4 (7.8)	62.4 (7.4)	62.2 (6.9)	<0.001
Sex					0.008
Men	1217 (46.4%)	461 (47.3%)	374 (52.1%)	185 (53.5%)	
Women	1405 (53.6%)	514 (52.7%)	344 (47.9%)	161 (46.5%)	
Income					<0.001
Low	776 (30.0%)	310(32.1%)	256(35.9%)	121 (35.3%)	
Medium	724 (28.0%)	304(31.5%)	232(32.5%	136 (39.7%)	
High	1090 (42.1%)	351(36.4%)	225(31.6%)	86 (25.1%)	
Education					0.003
Low	1681 (73.0%)	625 (68.2%)	486 (72.5%)	256 (80.5%)	
Medium	333 (14.5%)	161 (17.6%)	93 (13.9%)	36 (11.3%)	
High	290 (12.6%)	130 (14.2%)	91 (13.6%)	26 (8.2%)	
Urbanization					<0.001
Low	648 (24.7%)	244 (25.0%)	181 (25.2%)	110 (31.8%)	
Medium	634 (24.2%)	295 (30.3%)	245 (34.1%)	124 (35.8%)	
High	1340 (51.1%)	436 (44.7%)	292 (40.7%)	112 (32.4%)	
Smoking					<0.001
Non smoker	1837 (70.2%)	656 (67.5%)	442 (61.6%)	197 (57.1%)	
Ex-smokers	84 (3.2%)	34 (3.5%)	27 (3.8%)	25 (7.2%)	
Current smokers	695 (26.6%)	282 (29.0%)	248 (34.6%)	123 (35.7%)	
Survey year					<0.001
1997	1219 (46.5%)	379 (38.9%)	318 (44.3%)	136 (39.3%)	
2000	462 (17.6%)	159 (16.3%)	107 (14.9%)	69 (19.9%)	
2004	621 (23.7%)	241 (24.7%)	173 (24.1%)	71 (20.5%)	
2006	320 (12.2%)	196 (20.1%)	120 (16.7%)	70 (20.2%)	
Alcohol drinking	737 (28.7%)	308 (32.2%)	256 (36.2%)	131 (38.4%)	<0.001
Physical activity (MET/week)	79.2 (90.8)	89.8 (102.5)	100.2 (110.3)	121.7 (115.8)	<0.001
BMI (kg/m^2^)	23.2 (3.7)	23.1 (3.6)	22.7 (3.6)	22.2 (3.2)	<0.001
BMI>24 (kg/m^2^)	967 (39.9%)	330 (36.6%)	223 (32.9%)	81 (24.5%)	<0.001
Energy intake (kcal/day)	2038.2 (613.6)	2103.8 (617.0)	2160.0 (621.2)	2342.5 (711.8)	<0.001
Fat intake (g/day)	66.2 (36.4)	67.0 (35.5)	66.6 (36.7)	70.1 (41.6)	0.32
Protein intake (g/day)	62.9 (23.2)	63.6 (21.7)	63.8 (21.8)	68.4 (27.5)	<0.001
Carbohydrate intake (g/day)	292.4 (103.1)	305.3 (104.8)	320.2 (109.6)	347.7 (110.2)	<0.001
Traditional southern dietary pattern score	−0.2 (0.9)	−0.0 (0.9)	0.1 (0.8)	0.2 (0.9)	<0.001
Modern dietary pattern score	0.0 (0.9)	−0.1 (0.8)	−0.2 (0.7)	−0.4 (0.6)	<0.001
Chili intake (g/day)	0.0 (0.0)	9.8 (5.4)	33.5 (8.4)	75.3 (23.8)	<0.001
Hypertension	948 (38.2%)	311 (33.9%)	215 (31.3%)	92 (27.8%)	<0.001
Diabetes	89 (3.5%)	33 (3.4%)	22 (3.1%)	5 (1.5%)	0.260
Stroke	62 (2.4)	26 (2.7%)	6 (0.8%)	6 (1.8%)	0.046
Self-reported poor memory	561(21.5%)	189(19.6%)	116(16.4%)	98(28.6%)	<0.001
Self-reported memory decline	998(39.3%)	353(37.0%)	260(37.2%)	168(50.0%)	<0.001

^1^ Data are presented as mean (SD) for continuous measures, and n (%) for categorical measures.

**Table 2:** Regression coefficients (95% CI) for cognitive function by quartiles of chili intake among Chinese adults aged 55 years and above attending China Health and Nutrition Survey (*n* = 4852) ^1^.

	None	1–20	20–50	>50	*p* value
Global cognitive function					
Model 1	0.00	0.16 (−0.15–0.47)	−0.60 (−0.96–0.24)	−1.87 (−2.39–1.35)	<0.001
Model 2	0.00	0.27 (−0.05-0.58)	−0.27 (−0.64–0.10)	−1.10 (−1.62–0.57)	0.001
Model 3	0.00	0.28 (−0.04-0.60)	−0.26 (−0.63–0.12)	−1.12 (−1.64–0.59)	0.001
Model 4	0.00	0.18 (−0.14-0.51)	−0.36 (−0.74–0.02)	−1.17 (−1.70–0.63)	<0.001
Model 5	0.00	0.17 (−0.18-0.52)	−0.31 (−0.72–0.10)	−1.13 (−1.71–0.54)	0.001

^1^ Model 1 Model 1 adjusted for age, gender and energy intake. Model 2 further adjusted for intake of fat, smoking, alcohol drinking, income, urbanicity, education, and physical activity. Model 3 further adjusted for overall dietary patterns. Model 4 further adjusted for BMI and hypertension. All the adjusted variables are treated as time-varying covariates (except gender). Model 5 further excluded those who only participated in one wave of the cognitive function tests.

**Table 3:** Odds ratios (95% CI) for self-reported poor memory, self-reported memory decline, and global cognitive score below 7 by levels of chili intake among Chinese adults aged >= 55 years old by characteristics, China Health and Nutrition Survey (*n* = 4852) ^1^.

	None	1–20	20–50	>50	*p* value
Self-reported poor memory					
Model 1	1.00	1.17 (1.02–1.34)	1.07 (0.91–1.26)	2.26 (1.82–2.81)	<0.001
Model 2	1.00	1.14 (0.98–1.32)	1.02 (0.85–1.21)	2.03 (1.61–2.56)	<0.001
Model 3	1.00	1.13 (0.97–1.31)	1.00 (0.84–1.19)	1.98 (1.56–2.50)	<0.001
Model 4	1.00	1.23 (1.05–1.43)	1.06 (0.89–1.27)	2.17 (1.69–2.77)	<0.001
Model 5	1.00	1.20 (1.02–1.42)	1.10 (0.90–1.33)	2.12 (1.63–2.77)	0.001
Self-reported memory decline					
Model 1	1.00	1.07 (0.96–1.20)	1.13 (0.99–1.29)	1.75 (1.45–2.11)	<0.001
Model 2	1.00	1.05 (0.93–1.20)	1.10 (0.95–1.28)	1.61 (1.31–1.99)	<0.001
Model 3	1.00	1.04 (0.92–1.18)	1.08 (0.93–1.25)	1.54 (1.25–1.90)	<0.001
Model 4	1.00	1.06 (0.93–1.21)	1.10 (0.95–1.29)	1.56 (1.26–1.94)	0.001
Model 5	1.00	1.08 (0.93–1.24)	1.12 (0.95–1.33)	1.56 (1.23–1.97)	0.001

^1^ Model 1 Model 1 adjusted for age, gender and energy intake. Model 2 further adjusted for intake of fat, smoking, alcohol drinking, income, urbanicity, education, and physical activity. Model 3 further adjusted for overall dietary patterns. Model 4 further adjusted for BMI and hypertension. Model 5 further excluded those who only participated in one wave of the cognitive function tests. All the adjusted variables are treated as time-varying covariates (except gender).

## Supplementary Materials

The following are available online at https://www.mdpi.com/2072-6643/11/5/1183/s1, Figure S1: Prevalence (%) of self-reported poor memory and self-reported memory decline by year and chili intake among Chinese adults aged>=55 years and attended at least two waves of cognition tests.
