# Supplementary material for: High Chili Intake and Cognitive Function among 4582 Adults: An Open Cohort Study over 15 Years
Source: Nutrients. 2019 May 27;11(5):1183. doi: 10.3390/nu11051183 (PMC6566199; doi:10.3390/nu11051183)

Figure S1 Prevalence (%) of self-reported poor memory and self-reported memory decline by year and chili intake among Chinese adults aged>=55 years and attended at least two waves of cognition tests

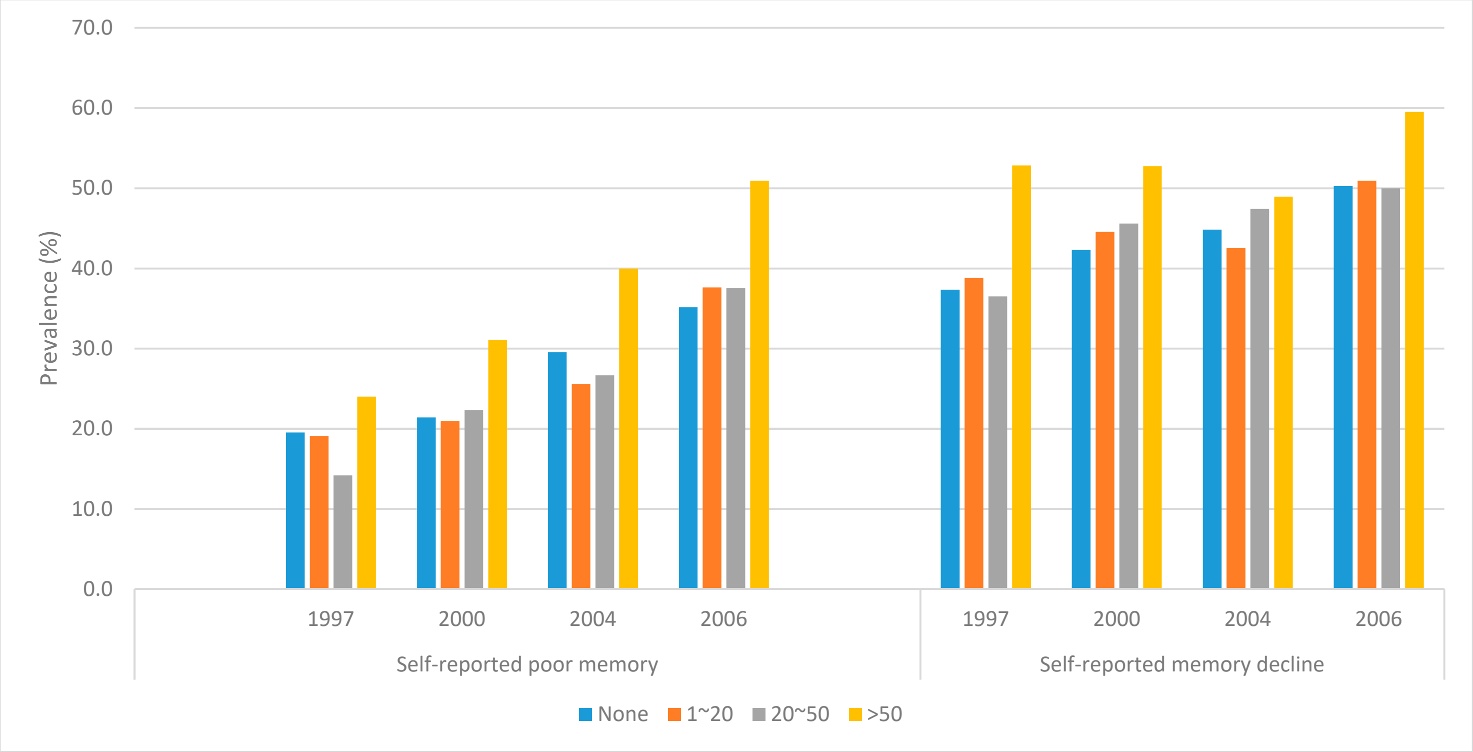

Supplement: Supplementary file 1 [file nutrients-11-01183-s001.pdf]
